# Dynamic Regulation of Gonadal Transposons and Pseudogenes via PIWI/piRNA Pathway in Gynogenetic Japanese Flounder (*Paralichthys olivaceus*)

**DOI:** 10.3390/biology14101464

**Published:** 2025-10-21

**Authors:** Zeyu Liu, Weigang Li, Fengchi Wang, Wei Lu, Fan Yang, Qingke Zhang, Jie Cheng

**Affiliations:** 1 Key Laboratory of Marine Genetics and Breeding (Ministry of Education), College of Marine Life Sciences, Ocean University of China, Qingdao 266003, China; 2 Key Laboratory of Tropical Aquatic Germplasm of Hainan Province, Sanya Oceanographic Institution, Ocean University of China, Sanya 572024, China; 3 Shandong Key Laboratory of Marine Seed Industry, Ocean University of China, Qingdao 266003, China; 4 Laboratory for Marine Fisheries Science and Food Production Processes, Qingdao Marine Science and Technology Center, Qingdao 266237, China

**Keywords:** PIWI/piRNA pathway, gynogenesis, *Paralichthys olivaceus*, transposon, pseudogenes

## Abstract

PIWI-interacting RNAs (piRNAs) are small non-coding RNAs that interact with PIWI proteins and play essential roles in genome stability, gonadal development, and gametogenesis in animals. The Japanese flounder (*Paralichthys olivaceus*) is an important marine culture teleost in North Asia, showing pronounced sexual size dimorphism, where gynogenetic induction of all-female cohorts can markedly enhance production. However, the PIWI/piRNA pathway in gynogenetic diploid *P. olivaceus*, which often exhibit gonadal dysgenesis, poor gamete quality, and low fertilization rates, remains poorly understood. In this study, RNA-seq and small RNA-seq data from 11 tissues and 6 developmental stages of common *P. olivaceus*, as well as the gonads of gynogenetic *P. olivaceus,* were analyzed to characterize the PIWI/piRNA pathway and its roles in transposon and gene regulation within the germline. The results showed that PIWI/piRNA genes were predominantly expressed in gonads and early embryogenesis in common *P. olivaceus*, with the highest expression in testis. Clustered piRNAs were identified in the testis and early embryos of common *P. olivaceus*, which targeted multiple transposon and gene families. Intriguingly, gynogenetic *P. olivaceus* gonads harbored abundant clustered piRNAs not only in the testes but also in the ovaries, both targeting similar transposon families as that in common *P. olivaceus*. Notably, the DNA transposon *Tc1/Mariner* family and *pim* genes were the most heavily targeted by piRNAs in gynogenetic *P. olivaceus*, with testis-biased expression. Expanded *pim* genes were identified in *P. olivaceus*, overlapping with piRNA clusters, and the *in vitro* test in *P. olivaceus* testes revealed that the expanded *pim* genes may be pseudogenes as a piRNA cluster reference to generate piRNAs regulating the conventional *pim* members. These unique features of the PIWI/piRNA pathway in gynogenetic diploid *P. olivaceus* may underline their impaired reproductive ability, and have important theoretical and practical implications for teleost gynogenetic breeding.

## Simple Summary

To characterize the PIWI/piRNA pathway and its role in transposon and gene regulation within the germline, RNA-seq and small RNA-seq data were analyzed from different tissues and developmental stages of common *P. olivaceus*, as well as the gonads of gynogenetic *P. olivaceus*, often exhibiting gonadal dysgenesis, poor gamete quality, and low fertilization rates. Clustered piRNAs were mainly detected in the testes and early embryos of common *P. olivaceus*, whereas the ovaries of gynogenetic *P. olivaceus* harbored abundant testis-like piRNA clusters, representing a marked divergence from common *P. olivaceus* and suggesting that piRNA-mediated regulation may play a distinct role in the gynogenetic lineage. In gynogenetic *P. olivaceus*, *pim* genes were heavily targeted by piRNAs, exhibiting male-biased expression and conspicuous expansion across the teleost lineage. Expanded *pim* genes in *P. olivaceus* gonads overlapped with piRNA clusters, and the *in vitro* assay showed that overexpression of an expanded *pim*-related piRNA cluster significantly reduced the expression of conventional *pim*s in *P. olivaceus* testes, supporting the model that the expanded *pim* genes may be putative pseudogenes as a piRNA cluster reference to generate piRNAs regulating the conventional *pim* members.

## 1. Introduction

PIWI-interacting RNAs (piRNAs) are a class of 24–32 nt non-coding small RNAs that associate with PIWI proteins to form PIWI-piRNA complexes and play essential roles in genome integrity maintenance, gametogenesis, and heterochromatin biogenesis [1]. piRNA biogenesis occurs via two complementary pathways. In the primary pathway, long single-strand precursor transcripts are generated from discrete genomic loci known as piRNA clusters (piRCs) [2]. These precursors are endonucleolytically processed to yield 5′-monophosphorylated pre-piRNAs, together with the Argonaute protein and subsequent 3′-end trimming, to produce mature piRNAs [3,4,5,6]. In the secondary (ping-pong) pathway, secondary pre-piRNAs arise from reciprocal cleavage events between antisense and sense transcripts. PIWI-piRNA complexes recognize and slice complementary transposon-derived RNAs, generating 5′-monophosphorylated fragments and, in turn, guide cleavage of piRNA cluster transcripts. This ping-pong cycle amplifies both sense and antisense piRNA populations [7,8]. Cleavage by PIWI at the 10th nucleotide of the target imparts a characteristic sequence signature: primary piRNAs exhibit a strong bias for uridine at their 5′ end (position 1), whereas secondary piRNAs preferentially contain adenine at position 10 [8]. Consequently, piRNA loci typically display clustered organizations reflecting their ping-pong-dependent amplification [9].

Other than PIWI proteins, the full functionality of the PIWI/piRNA pathway requires the participation of numerous auxiliary factors, such as the Tudor domain-containing proteins (TDRDs), the mitochondrial membrane proteins GPAT2 and MitoPLD, and the DEAD-box helicase VASA [10,11,12]. The earliest functional characterizations of this pathway is centered on its role in transposon silencing: piRNA complexes recognize complementary transposon transcripts and, via PIWI-mediated endonucleolytic cleavage, suppress transposon expression to maintain genomic stability [13]. Loss of the piRNA granule protein Asz1 in zebrafish (*Danio rerio*) leads to a profound depletion of germ cells and the failure of gonadal development, accompanied by transposable element (TE) de-repression and disorganization of piRNA granules, indicating that the piRNA pathway is essential for TE silencing and the maintenance of germline homeostasis [14]. Meanwhile, in several African cichlids, piRNAs in gonads and early developmental stages evolve rapidly and exhibit stronger targeting of more active transposable element families, revealing a dynamic co-evolutionary relationship between the host repression pathway and transposons [15]. Furthermore, emerging evidence indicates that the piRNA pathway contributes to gene regulation. In the spinyhead croaker (*Collichthys lucidus*), numerous sex-biased piRNAs and piRNA clusters have been identified and are predicted to target gonadal development-related genes such as *dazl*, *amh*, and *piwil1*, suggesting that piRNAs may influence gonadal development through the modulation of gene expression [16].

In addition to their well-established roles in male organisms, piRNAs also exhibit diverse functions in females. For instance, other than in testes, abundant piRNAs are also present in *Drosophila* ovaries, and mutations in the PIWI/piRNA pathway result in female sterility. This phenotype is closely associated with the pathway’s roles in transposon silencing and regulation of gene expression [17]. In mammals, the PIWI/piRNA pathway also plays essential roles during oogenesis. For instance, piRNAs in mice can mediate the inactivation of the *Astrin* factor, thereby impairing both mitosis and meiosis in oocytes, ultimately affecting female reproductive development [18]. In teleosts, up-regulation of the hypothalamic–pituitary–gonadal (HPG) axis has been observed in reproductively active female crucian carp (*Carassius auratus*), accompanied by down-regulation of the PIWI/piRNA pathway. This suppression reduces the silencing of reproduction-related genetic elements and is thought to facilitate ovulation [19].

The Japanese flounder (*Paralichthys olivaceus*) is an economically important marine fish that is widely distributed along the coast of China, Japan, and the Korean Peninsula. It has an XX/XY sex determination system with sex-reversed pseudo-males induced under environmental influences such as water temperature and external hormone. In addition, *P. olivacues* display pronounced sexual size dimorphism, with females typically attaining greater body size than males. Therefore, production of all-female cohorts via gynogenetic induction offers substantial economic benefits. However, current gynogenetic breeding methods suffer from several limitations, such as low fertilization rates, impaired gonadal development, and poor gamete quality [20]. These pathological phenotypes markedly constrain the practical application of gynogenetic breeding in *P. olivaceus*.

The PIWI/piRNA pathway plays pivotal roles in the germline, regulating gonadal development, gametogenesis, and related processes. Recent studies have indicated that both PIWI/piRNA [21,22,23] and steroidogenic [24] pathways play essential roles in *P. olivaceus* gonad development and gametogenesis, through ncRNA processing like clustered piRNAs [23] and miRNAs [25,26]. Characterizing the features and regulatory patterns of the PIWI/piRNA pathway in both common and gynogenetic *P. olivaceus* may yield novel strategies for all-female breeding. In this study, with RNA-seq and small RNA-seq data from 11 tissues and 6 developmental stages of common *P. olivaceus*, as well as the testes and ovaries of gynogenetic *P. olivaceus*, the PIWI/piRNA pathway components were characterized, and their roles in transposon and gene regulation within the germline were investigated. These results elucidate the fundamental characteristics of the PIWI/piRNA pathway in *P. olivaceus* and provide a foundation for further mechanistic studies and the optimization of gynogenetic breeding protocols.

## 2. Materials and Methods

### 2.1. Samples Used in This Study

The 1.5-year-old *P. olivaceus* were obtained from a commercial aquaculture facility in Qingdao, Shandong, China. We previously generated gynogenetic diploid *P. olivaceus* via hydrostatic pressure treatment, such that the ultraviolet light-irradiated *P. olivaceus* semen was inseminated into eggs from one mature female for 2 min of fertilization, and the eggs were used to induce a meiogynogenetic diploid by hydrostatic pressure of 60 MPa for 6 min to inhibit second polar body extrusion [25,27]. Sex-reversed gynogenetic pseudo-males were induced through high-temperature incubation during the sex differentiation period, and both gynogenetic diploid female and pseudo-males were raised to 1.5 years old for subsequent sequencing.

### 2.2. Transcriptome Data Analysis

The mRNA and small RNA transcriptomes used in this study included gonad samples from three pseudo-males and three females of 1.5-year-old gynogenetic *P. olivaceus* [25], as well as 11 tissues (brain, gill, heart, kidney, liver, intestine, stomach, muscle, spleen, testis, and ovary) of 1.5-year-old common *P. olivaceus* (pooled from three females and males for each tissue) and 6 developmental stages (pooled from 10 larvae for each stage), as follows:

Stage 1: 2-cell to 1K-cell through the morula (mulberry) stage;

Stage 2: Gastrula (anterior, middle, and posterior phases) and somitogenesis (anterior, middle, and posterior phases);

Stage 3: Hatching period and 1–2 days post hatch;

Stage 4: Pre-metamorphosis (12 days post hatch);

Stage 5: Metamorphosis phase 1 (initiation) and phase 2 (onset of morphological change);

Stage 6: Metamorphosis phase 3 (eye migration), phase 4 (late stage), and phase 5 (completion of settlement).

Total RNA was extracted from tissues and larvae using TRIZOL (Invitrogen, Waltham, MA, USA), treated by DNase I. The quality and quantity of RNA were checked on agarose gel and Nanodrop spectrophotometer. The Illumina TruSeq mRNA Stranded Sample Preparation Kit (Illumina, San Diego, CA, USA) was used to construct libraries according to the manufacturer’s protocol. The libraries were sequenced on Illumina HiSeq 2500 (Illumina, San Diego, CA, USA) using a 150 bp paired-end sequencing module. The raw RNA-seq read quality was assessed using FastQC v 0.11.9 [28], and adapter removal and quality trimming (Phred score ≥ 20) were performed with Trimmomatic v 0.38 [29]. The filtered clean reads were aligned to the reference *P. olivaceus* genome (GCF_001970005.1) using HISAT2 v 2.2.0 [30], and transcript abundances were estimated by StringTie v 2.0.6 [31], with FPKM values used as the expression metric. Weighted gene co-expression network analysis (WGCNA) [27] was conducted with the 11 tissue transcriptomes to identify the gonad-specific gene co-expression modules with the PIWI/piRNA pathway.

### 2.3. Small RNA Sequencing Data Analysis

Small RNA libraries were constructed using the TruSeq Small RNA sample Prep Kit (Illumina, San Diego, CA, USA), and were run on the Illumina HiSeq 2500 sequencer (Illumina, San Diego, CA, USA) with SE50. Raw reads from small RNA sequencing were first assessed by a quality check using FastQC v 0.11.9 [28], and Trimmomatic v 0.38 [29] was employed for quality trimming and adapter removal, with the following settings: minimum Phred quality score of 20, removal of adapter sequences, and retention of reads between 10 and 35 nt in length. The resulting high-quality reads were carried forward for downstream analyses. Redundancy reduction and low-complexity filtering of the trimmed small RNA libraries were performed using the NGS Toolbox suite [32]. Specifically, the TBr2_collapse.pl script was used to collapse redundant reads, retaining only unique sequence entries, and the TBr2_duster.pl script was applied to remove low-complexity sequences, both run with default parameters. Finally, global profiling of small RNA read counts by sequence length was generated using TBr2_basic-analyses.pl, using the default settings.

### 2.4. Annotation and Ping-Pong Signature of Small RNAs

Annotation and classification of small RNA species were performed using unitas v 1.7.7.1 [33]. Reference sequence libraries for microRNA (miRNA), transfer RNA (tRNA), ribosomal RNA (rRNA), and messenger RNA (mRNA) were constructed. We combined our laboratory-sequenced and annotated *P. olivaceus* miRNA, tRNA, and mRNA datasets with the pre-packaged reference libraries for the closely related turbot (*Scophthalmus maximus*) provided by unitas. Trimmed reads were mapped to the *P. olivaceus* genome (GCF_001970005.1) using sRNAmapper v 1.0.4 [32], and the resulting alignment files were supplied to unitas for annotation. The –pp option in unitas was used to analyze ping-pong signature profiles for each small RNA class. Reads that did not match any known RNA category (labeled “no-annotation”) were considered candidate piRNAs. Finally, the ping-pong cycle characteristics of the small RNA dataset were further examined using PPmeter v 0.4 [34], with all software run under default parameter settings.

### 2.5. Prediction and Annotation of piRNA Cluster

piRNA cluster prediction was performed using proTRAC v 2.4.4 [35]. First, piRNA sequences annotated by unitas were aligned to *P. olivaceus* genome with sRNAmapper (–e 2 –f 6 –alignments best), and the resulting alignment files, together with the NCBI *P. olivaceus* genome assembly, RepeatMasker v 4.0.0 [36] output, and gene annotation GFF, were supplied to proTRAC for cluster calling. ProTRAC parameters were set to a minimum cluster length of 1000 nt, a maximum individual piRNA length of 34 nt, and a significance threshold of *p* ≤ 0.05 per kilobase. This analysis yielded the number, length, sequence content, and genomic coordinates of predicted piRNA clusters (piRCs). Finally, predicted piRNA clusters were compared against the RepeatMasker-annotated transposon sequences using NCBI BLAST+ v 2.11.0 [37] (*p* < 1 × 10^−5^, score > 200). Genome-wide transposon annotation in *P. olivaceus* genome was conducted with RepeatMasker. For each piRNA cluster, the highest-scoring hit was retained for transposon targets annotation. Gene Ontology (GO) enrichment was also conducted using Omicshare tools [38].

### 2.6. Identification and Characterization of piRNA-Associated TEs and Genes

piRNA sequences predicted from the gonads of gynogenetic *P. olivaceus* were aligned to the *P. olivaceus* transposon and mRNA libraries using Bowtie2 v 2.4.4 [39] with default parameters (–end-to-end –very-sensitive –N 1 –L 15 –a –seed 42). From the alignment results, we quantified, for each transposon family and mRNA, the number of mapped piRNAs to generate a profile of piRNA–transposon and piRNA–gene interactions in gynogenetic *P. olivaceus* gonads.

### 2.7. Identification and Characterization of Tc1/Mariner Transposon in P. olivaceus

To establish a *P. olivaceus*-specific *Tc1/Mariner* transposon database, we retrieved *Tc1/Mariner* sequences from Repbase [40] for the pufferfish *Takifugu rubripes* and from the literature on five additional teleosts—Atlantic cod (*Gadus morhua*, Gmo), green spotted puffer (*Tetraodon nigroviridis*, Tni), medaka (*Oryzias latipes*, Ola), three-spined stickleback (*Gasterosteus aculeatus*, Gac), and Nile tilapia (*Oreochromis niloticus*, Oni) [41]. These sequences were aligned to the *P. olivaceus* genome (GCF_001970005.1), and each matching locus plus its 1 000 bp upstream and downstream flanking regions were extracted. Open reading frames (ORFs) were predicted using NCBI’s ORF finder [42], and sequences harboring ORFs encoding peptides longer than 200 amino acids were retained to compose the *P. olivaceus Tc1/Mariner* transposon library.

Amino acid sequences of the *P. olivaceus Tc1/Mariner* transposase proteins were aligned with homologous transposase sequences retrieved from NCBI and from the literature [41] using Clustal W v 2.0. A phylogenetic tree was constructed in MEGA 7 [43] by the Maximum Likelihood method under the Jones–Taylor–Thornton (JTT) substitution model, with bootstrap support of 1000 replicates. Protein domain architecture was predicted via the SMART web server v 9.0 [44]. To delineate the inverted terminal repeats (ITRs) and coding region domains of *P. olivaceus Tc1/Mariner* elements, the *P. olivaceus* transposon library was aligned in MEGA 7 against structurally characterized *Tc1/Mariner* sequences (Passport_Gac, Frog_Pince_Gac, pogo_Gac, Minos_Gmo, Sleeping Beauty_Tru, and Bari_Tni) reported in the literature [41], and domain predictions from SMART (https://smart.embl.de/smart/change_mode.cgi, accessed on 20 October 2020) were integrated to confirm ITR boundaries and conserved catalytic and DNA-binding motifs. Subsequently, one representative member from each subfamily identified in *P. olivaceus* was selected for expression analysis by qRT-PCR.

### 2.8. Identification and Characterization of Pim Genes and Associated piRNAs in P. olivaceus

Using the serine/threonine kinase *pim* gene annotated in the *P. olivaceus* genome (GCF_001970005.1) as a reference, 14 members of the *pim* family were identified. Amino acid sequences of PIM from humans (*Homo sapiens*), mice (*Mus musculus*), Amazon mollies (*Poecilia formosa*), and barramundi (*Lates calcarifer*) were retrieved from NCBI and Ensembl (http://asia.ensembl.org/index.html, accessed on 12 October 2020). These sequences, together with the *P. olivaceus pim* members, were aligned using Clustal W in MEGA 7 [43]. A phylogenetic tree was constructed by the Maximum Likelihood method, with the best-fit model determined as Jones–Taylor–Thornton (JTT) + Gamma distributed with Invariant sites (G + I), and bootstrap support of 1000 replicates. The expression of *pim* genes and their targeted piRNAs in *P. olivaceus* was analyzed using transcriptome data and qRT-PCR. Due to sequence similarity among family members, *pim* genes with greater sequence divergence and their corresponding targeted piRNAs were selected. Potential piRNA clusters (piRCs) were identified by aligning long non-coding RNA (LncRNA) transcriptome sequencing data [25] from *P. olivaceus* with piRNAs and *pim* genes, to explore the regulatory relationships in a piRC–piRNA–gene network.

### 2.9. RNA Extraction and qRT-PCR

Total RNA from gonad tissues was extracted using the TRIzol reagent. qRT-PCR was carried out on a LightCycler 480 real-time PCR system (Roche, Basel, Switzerland). For mRNA targets, the reaction was 95 °C for 5 min, followed by 40 cycles of 95 °C for 15 s and 60 °C for 45 s. For small RNA targets, the reactuib was 95 °C for 5 min, followed by 40 cycles of 95 °C for 15 s and 60 °C for 20 s. Raw Ct values were obtained and relative expression levels calculated using the 2^−ΔΔCt^ method [45]. Statistical significance was assessed by an independent-samples *t*-test, with *p* < 0.05 considered significant (*) and *p* < 0.01 considered highly significant (**). qRT-PCR primers were designed using Primer5 and listed in Table S1. The *Actb* and *Ubce* genes were employed as internal reference controls for mRNA [46], and miR-22-3p and miR-23a-3p for small RNA [47]. The reverse small RNA primer was the mRQ 3′ primer from the Mir-X^TM^ miRNA First-Strand Synthesis Kit (Clontech, Mountain View, CA, USA).

### 2.10. In Vitro Overexpression of piRC

The piRNA cluster piRC_00046184 was amplified and cloned into the pEGFP-C1 vector by In-Fusion Cloning using the primers listed in Table S1. The recombinant pEGFP-piRC184 plasmids were verified by sequencing. pEGFP-piRC184 were in vitro overexpressed in the testis tissue cultures of 1.5-year-old *P. olivaceus* (688.0 ± 17.0 g; 37.9 ± 0.5 cm) as described by Wang et al. [26]. The testis samples were cut into blocks and placed on nitrocellulose membranes upon 1.5% agarose gel, and then cultured in 800 μL of L-15 medium supplemented with 0.5% FBS and 5% penicillin–streptomycin–amphotericin B in 12-well plates. The testis blocks were transfected with 1 μg pEGFP-piRC184/pEGFP-C1 plasmids, respectively, and 2 μL Lipofectamin^TM^ 3000 (Thermo Fisher Scientific Inc., Waltham, MA, USA) transfection reagent diluted with 50 µL L-15 medium with 0.5% FBS and 5% penicillin–streptomycin–amphotericin B solution. After 72 h of transfection, testis blocks were sampled for RNA extraction and qRT-PCR.

## 3. Results

### 3.1. Expression of PIWI/piRNA Pathway Genes in P. olivaceus

**Figure 1:**
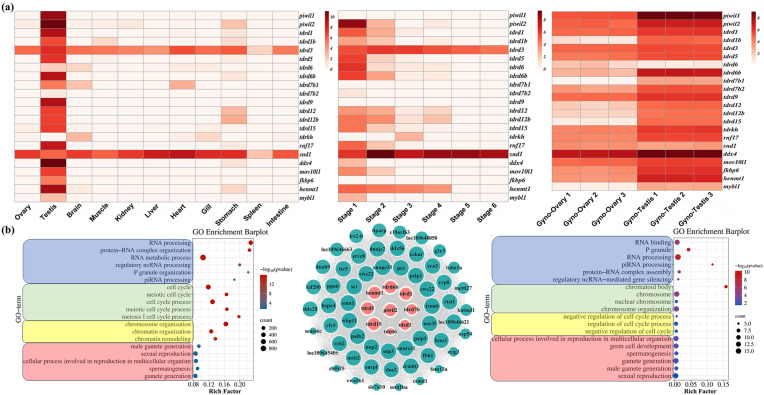
The regulatory co-expression of PIWI/piRNA pathway genes in *P. olivaceus*. (**a**) PIWI/piRNA pathway gene expression illustrated as log_2_(RPKM + 1) values in common *P. olivaceus* tissues, developmental stages, and gynogenetic *P. olivaceus* gonads; (**b**) functional enrichment and gene network of PIWI/piRNA pathway genes in the testis-specific gene co-expression module from WGCNA. The left GO terms were enriched from all genes in the testis-specific module which contained the PIWI/piRNA genes, while the right GO terms were enriched from the top interactive genes with the PIWI/piRNA genes. It represented the top 10 genes (green) having the highest connectivity with the PIWI/piRNA genes (red) in the network.

The expression of PIWI/piRNA pathway genes were quantified across 11 adult tissues and six developmental stages of common *P. olivaceus*, as well as in the testes and ovaries of 1.5-year-old gynogenetic *P. olivaceus* (Figure 1a). Most PIWI/piRNA pathway genes were predominantly expressed in the testes of common *P. olivaceus* (Figure 1a), consistent with their essential roles in spermatogenesis and testicular development. In addition, most PIWI/piRNA genes were highly expressed during early development stages (Stages 1–2), with transcript levels declining sharply after hatching (Figure 1a). In gynogenetic *P. olivaceus*, PIWI/piRNA genes were highly expressed in testes, mirroring the patterns observed in common *P. olivaceus* (Figure 1a); however, they also showed high expression in ovaries, indicating the dynamic activity of PIWI/piRNAs in gynogenetic *P. olivaceus* ovaries. Subsequently, WGCNA was performed using the tissue transcriptomes, which identified testis-specific gene co-expression module based on the overrepresentation of genes expressed in testis. Nine PIWI/piRNA genes were found from the testis-specific module and enriched GO functions of reproduction and spermatogenesis, ncRNA processing, meiotic cell cycle, chromosome and chromatin organization, etc. (Figure 1b). The top 10 genes having the highest connectivity with these PIWI/piRNA genes also enriched functions of reproduction and spermatogenesis, ncRNA processing, meiotic cell cycle, and chromosome and chromatin organization (Figure 1b), which indicated their conserved function with model organisms.

### 3.2. Small RNA Annotation and Spatio-Temporal Distribution in P. olivaceus

**Figure 2:**
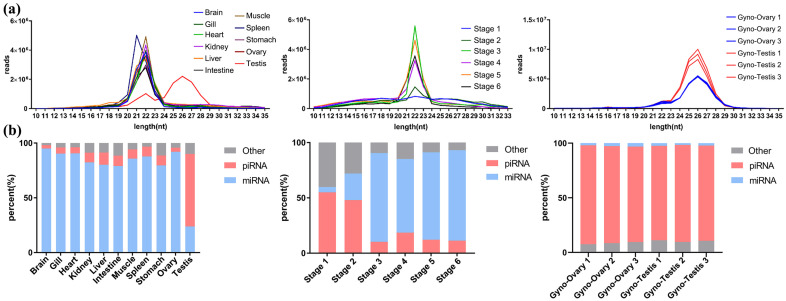
The abundance and diversity of small RNAs in *P. olivaceus*. (**a**) Total small RNA abundance in common *P. olivaceus* tissues, developmental stages, and gynogenetic *P. olivaceus* gonads; (**b**) classification of small RNA types in common *P. olivaceus* tissues, developmental stages, and gynogenetic *P. olivaceus* gonads.

After quality control of the small RNA sequencing datasets from 11 adult tissues and six developmental stages of common *P. olivaceus*, as well as the gonads of gynogenetic *P. olivaceus*, the read-length distributions were examined. In common *P. olivaceus* tissues, all tissues except the testes displayed a pronounced peak of read abundance in the miRNA size range (19–24 nt), while the testes showed a marked enrichment in the piRNA range (24–29 nt), indicating a higher piRNA content in the testes than in other tissues (Figure 2a). For developmental stages, the post-hatching stages (Stages 3–6) were dominated by miRNA-length reads (19–24 nt), whereas the pre-hatching stages (Stages 1–2) exhibited a more even distribution and substantial piRNA range abundance (Figure 2a), suggesting elevated piRNA levels during early embryogenesis. These length distribution patterns across tissues and stages mirrored the expression profiles of PIWI/piRNA pathway genes (Figure 1), jointly reflecting high PIWI/piRNA pathway activity and piRNA biogenesis in the testes and early embryos of common *P. olivaceus*. Interestingly, small RNA profiles from the gonads of gynogenetic *P. olivaceus* also revealed expected piRNA range (24–29 nt) read coverage in the testes, but female gonads also displayed abundant piRNA-length reads, with an overall distribution similar to the testes (Figure 2a), indicating high piRNA abundance in both the testes and ovaries of gynogenetic *P. olivaceus*. After collapsing redundant and low-complexity reads, small RNAs were further annotated. piRNAs constituted 59.2% of the annotated small RNAs in the testes of common *P. olivaceus*, whereas other tissues were dominated by miRNAs (Figure 2b). In the developmental stages, the proportion of piRNAs declined markedly over time, with the highest piRNA abundance observed in early embryogenesis (Stages 1–2) (Figure 2b). These findings correspond with the expression profiles of PIWI/piRNA pathway genes and the small RNA length distributions, indicating that piRNAs are predominantly present in the testes and during the early embryonic development of common *P. olivaceus*. In gynogenetic diploid *P. olivaceus*, both the testes and ovaries harbored substantial piRNA fractions (Figure 2b), in agreement with previous gene expression patterns (Figure 1a).

### 3.3. Ping-Pong Signature of P. olivaceus piRNAs

**Figure 3:**
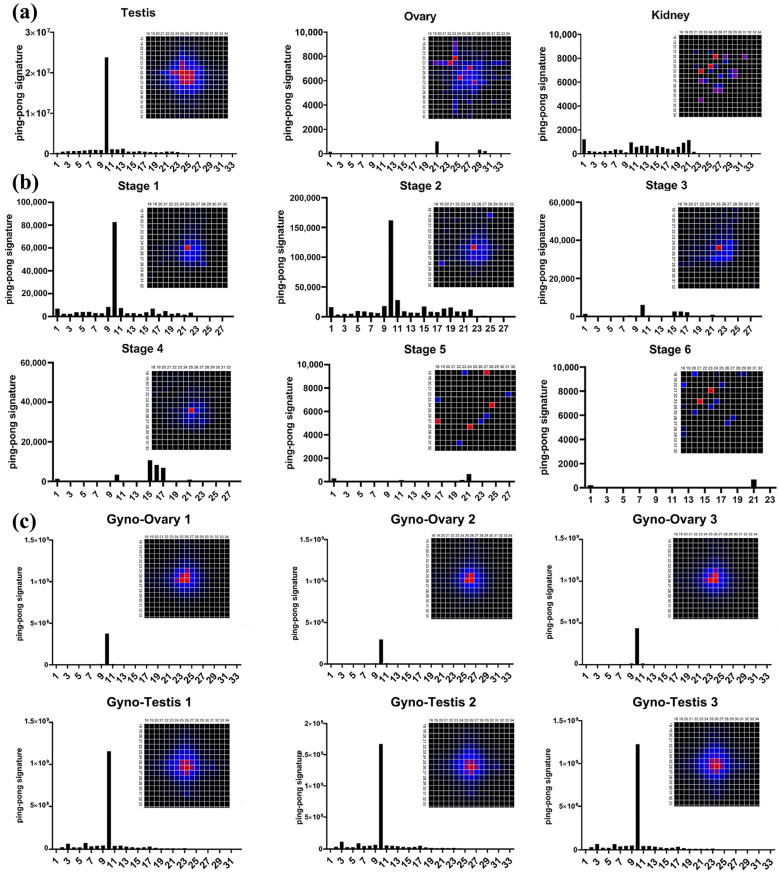
Ping-pong signature of piRNAs in (**a**) common *P. olivaceus* tissues, (**b**) common *P. olivaceus* developmental stages, and (**c**) gynogenetic *P. olivaceus* gonads. The bar plots represent the statistical significance index of the 10th nucleotide adenine enrichment (*p* value^−1^) of piRNAs, and the heat maps show the length distribution of piRNAs exhibiting ping-pong characteristics. The “×” symbol indicates the canonical 10-nt overlap characteristic of ping-pong amplification between sense and antisense piRNAs. The color intensity (black to red) indicates the frequency (minimum to maximum) of overlapping read pairs between sense and antisense piRNAs.

The ping-pong signature, a hallmark of secondary piRNA biogenesis, was assessed in representative tissues (testes, ovaries, kidneys) of common *P. olivaceus*. Only the testes exhibited a clear 10 nt overlap peak, with other tissues showing neither a strong signature nor comparable read counts (Figure 3a). Moreover, sequences exhibiting the ping-pong signature in the testes were mainly 23–27 nt in length, whereas the ovaries and kidneys displayed a dispersed length profile (Figure 3a). Across developmental stages, pronounced ping-pong signatures were confined to early embryogenesis (Stages 1–2), with the first four stages (Stages 1–4) showing a peak at 25 nt and later stages showing no clear enrichment (Figure 3b). These results indicated abundant secondary piRNAs, and thus potential piRNA cluster formation, in the testes and early embryos of common *P. olivaceus* (Figure 3b). In gynogenetic *P. olivaceus*, both the testes and ovaries displayed distinct 10 nt ping-pong peaks, with stronger signals in the testes (Figure 3c). Ping-pong-positive sequences in pseudo-male and female gonads were predominantly at 25–26 nt. Thus, both the ovaries and testes of gynogenetic *P. olivaceus* exhibited robust ping-pong signatures, reflecting extensive secondary piRNA biogenesis and likely piRNA cluster formation.

### 3.4. Characterization of piRNA Clusters in P. olivaceus

**Figure 4:**
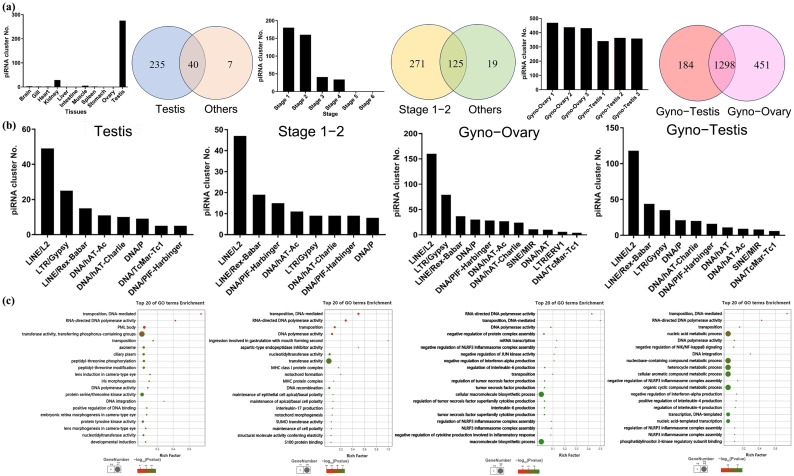
Identification and characterization of piRNA clusters in *P. olivaceus*. (**a**) The number and distribution of piRNA clusters in common *P. olivaceus* tissues, developmental stages, and gynogenetic *P. olivaceus* gonads; (**b**) the number of piRNA clusters indicating transposon families in common *P. olivaceus* tissues, developmental stages, and gynogenetic *P. olivaceus* gonads; (**c**) GO enrichment for piRNA cluster targeted genes in common *P. olivaceus* tissues, developmental stages, and gynogenetic *P. olivaceus* gonads.

piRNA clusters were identified based on piRNA sequences from 11 adult tissues and 6 developmental stages of common *P. olivaceus* (Table S2). Among the adult tissues, only the testes contained abundant identifiable piRNA clusters, while other tissues harbored few or none (Figure 4a). Across the developmental stages, the number of piRNA clusters showed a decreasing trend, with substantial clusters detected only during early embryogenesis (Stages 1–2) (Figure 4a). These findings are consistent with previous results regarding piRNA abundance (Figure 2) and the presence of ping-pong signatures (Figure 3). Further analysis comparing piRNA clusters revealed that the vast majority of clusters were testis-specific, with only a small number uniquely present in other tissues, and most clusters were enriched in the early stages (Stages 1–2) (Figure 4a). In gynogenetic *P. olivaceus*, a large number of piRNA clusters were identified in both the testes and ovaries, with the ovaries surprisingly containing even more clusters than the testes, a striking contrast to the pattern observed in common *P. olivaceus* (Figure 4a). Comparison of piRNA clusters between gynogenetic testes and ovaries showed that most clusters were shared between sexes, with only a small number being testis- or ovary-specific (Figure 4a). These results suggested that the ovaries of gynogenetic *P. olivaceus* harbor abundant piRNAs, many of which form clusters that are largely identical to those in the testes.

An essential function of piRNAs is to associate with TEs and regulate their expression. To explore the potential role of the PIWI/piRNA pathway in *P. olivaceus*, a total of four transposon classes, comprising 41 families and 270,142 copies, were identified in *P. olivaceus* genome, with the DNA transposons as the most abundant and diverse class, accounting for approximately 67% of all TEs (Table S3). Therefore, piRNA clusters from common *P. olivaceus* testes and embryonic stages (Stages 1–2) were mapped against the *P. olivaceus* transposon library. In the testes, piRNA clusters predominantly aligned to eight transposon families, with the highest hit counts for *LINE/L2*, *LTR/Gypsy*, and *LINE/Rex-Babar* (Figure 4b). Similarly, in stages 1 and 2, piRNA clusters associated most notably with *LINE/L2*, *LINE/Rex-Babar*, and *DNA/PIF-Harbinger*, paralleling the testis profile (Figure 4b). In gynogenetic *P. olivaceus*, testicular piRNA clusters aligned to ten transposon families, again with *LINE/L2*, *LINE/Rex-Babar*, and *LTR/Gypsy* as the most targeted (Figure 4b), while ovarian clusters associated similar *LINE/L2*, *LINE/Rex-Babar*, and *LTR/Gypsy*, and overall mirrored the testis pattern (Figure 4b). Other than transposons, many protein coding genes were also indicated by the piRNA clusters. The GO enrichment for piRNA-associated genes in common *P. olivaceus* testes included “transposition”, “DNA polymerase activity”, “protein serine/threonine kinase activity”, etc., while in embryonic stages 1 and 2, it enriched “transposition”, “DNA polymerase activity”, “transferase activity”, etc. (Figure 4c). In addition, the piRNA-associated genes in gynogenetic *P. olivaceus* ovaries enriched the GO functions of “transposition”, “DNA polymerase activity”, “mRNA transcription”, etc., while in the testes they enriched “transposition”, “DNA polymerase activity”, “nucleic acid metabolic process”, etc. (Figure 4c).

### 3.5. The piRNA-Targeted Tc1/Mariner Transposons in Gynogenetic P. olivaceus

**Figure 5:**
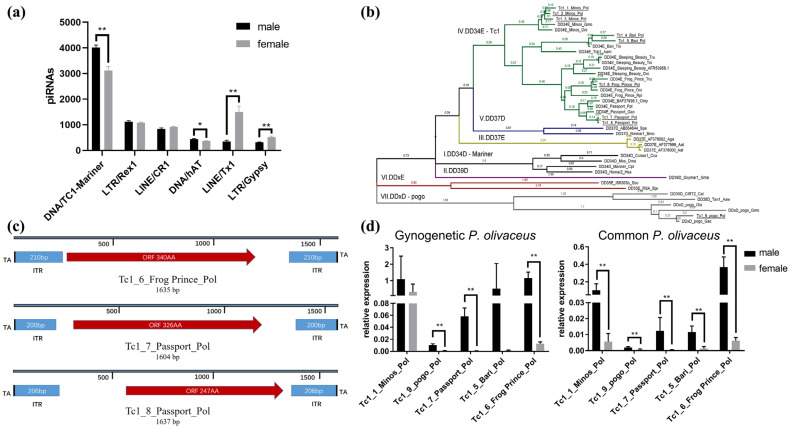
piRNA targeting transposons in gynogenetic *P. olivaceus* gonads. (**a**) The most piRNA-targeted TE families in gynogenetic *P. olivaceus* gonads; (**b**) phylogeny of *Tc1/mariner* family in *P. olivaceus* and other selected species. The seven branches of the *Tc1/mariner* family are represented by different colors. The representation of each transposon is as transposon type, name, and Latin name abbreviation of the species. For example, in “DD34E_Minos_Gmo”, “DD34E” refers to the type, “Minos” is the transposon subfamily, and “Gmo” is the abbreviation of the species “*Gadus morhua*”. The newly identified transposons in *P. olivaceus* are distinguished by the following lines. The number represents the self-test value of the tree branch that occurred in the phylogeny; (**c**) the structure of *Tc1/Mariner* transposons in *P. olivaceus*. The red box represents the start and end codons of ORF translation, and the blue box represents the end reverse repeat sequence (ITR); (**d**) qRT-PCR of *Tc1* members in gynogenetic and common *P. olivaceus* gonads. * indicates *p* < 0.05, ** indicates *p* < 0.01.

Due to the distinctive features of the PIWI/piRNA pathway in gynogenetic *P. olivaceus*, the piRNA-targeting transposons in both the testes and ovaries of gynogenetic *P. olivceus* were further investigated. The DNA transposon *Tc1/Mariner* family was the most heavily targeted by piRNAs (Figure 5a), suggesting a regulatory relationship between piRNAs and *Tc1/Mariner* elements. Nine *Tc1/Mariner* transposase ORFs encoding peptides longer than 200 amino acids were identified as members of the *P. olivaceus Tc1/Mariner* family (Table S4). Phylogenetic analysis incorporating *P. olivaceus* and other teleost *Tc1/Mariner* sequences grouped the *P. olivaceus* elements into five subfamilies: Passport-like, SB-like, Frog_Prince-like, Minos-like, and Bari-like (Figure 5b). Among the nine candidates, *Tc1_6_Frog_Prince_Pol*, *Tc1_7_Passport_Pol*, and *Tc1_8_Passport_Pol* contained discernible ITRs (Figure 5c), while *Tc1_6_Frog_Prince_Pol* and *Tc1_7_Passport_Pol* also harbored intact transposase domains and were classified as autonomous elements (Figure 5c). Consistent with the canonical *Tc1/Mariner* architecture, both full-length elements (*Tc1_6_Frog Prince_Pol* and *Tc1_7_Passport_Pol*) were flanked by TA dinucleotide target site duplications (Figure 5c and Table S4), which served as the recognition motifs for excision from the donor locus and integration into new genomic sites [48]. As qRT-PCR showed, most tested *Tc1* elements exhibited higher expression in testes than in ovaries in common and gynogenetic *P. olivaceus* (Figure 5d).

### 3.6. The piRNA-Targeted Pim Genes in Gynogenetic P. olivaceus

**Figure 6:**
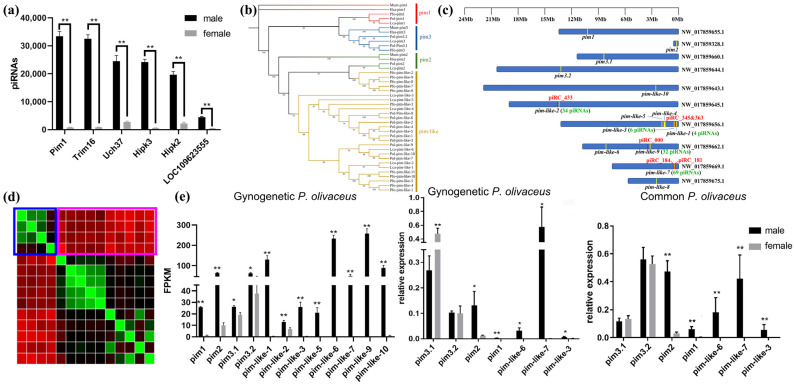
piRNA targeting genes in gynogenetic *P. olivaceus* gonads. (**a**) The most piRNA-targeted genes in gynogenetic *P. olivaceus* gonads; (**b**) phylogeny of the *pim* family in *P. olivaceus* and other selected species. The four branches of the *pim* family are represented by different colors. The number represents the self-test value of the tree branch that occurred in the phylogeny; (**c**) the co-localization of *pim* genes and piRNA clusters along the genomic scaffolds; the red characters represent the piRNA clusters, and the green characters represent the mapped piRNAs; (**d**) sequence similarity of conventional and expanded *pim* genes in *P. olivaceus*, colors from green to red represent increasing sequence divergence; (**e**) expression of *pim* genes in *P. olivaceus* gonads as transcriptome data of gynogenetic *P. olivaceus*; qRT-PCR of common and gynogenetic *P. olivaceus* gonads. * indicates *p* < 0.05, ** indicates *p* < 0.01.

The piRNA-targeting genes were also investigated in the gonads of gynogenetic *P. olivaceus* (Table S5). Serine/threonine kinase *pim* genes were the most heavily targeted by piRNAs (Figure 6a), suggesting a regulatory relationship between piRNAs and *pim* genes. In *P. olivaceus*, 14 members of the *pim* family were identified. Phylogenetic analysis revealed that mammals (humans and mice) possess only three conventional *pim* members (*pim1*, *pim2*, and *pim3*), whereas teleosts exhibit numerous expanded *pim*-like members, with clear divergence between the conventional and expanded types (Figure 6b,c). Sequence similarity of the *P. olivaceus pim* members further indicated substantial differences between the expanded and conventional *pim* genes (Figure 6d), suggesting that these two groups may perform diverged functions. Expression profiling showed that most *pim* members, particularly the expanded ones, display pronounced male-biased expression (Figure 6e). Genomic localization of piRNA clusters (piRCs), piRNAs, and *pim* genes revealed that many expanded *pim* genes are positioned close to, or even overlapped with, piRCs accompanied by numerous mapped piRNAs (Figure 6c). Collectively, these results suggested that the expanded *pim* genes in *P. olivaceus* may serve as putative templates for piRNA clusters, producing piRNAs that target other *pim* members (such as the conventional *pim*s) via sequence similarity, thereby mediating regulatory interactions.

**Figure 7:**
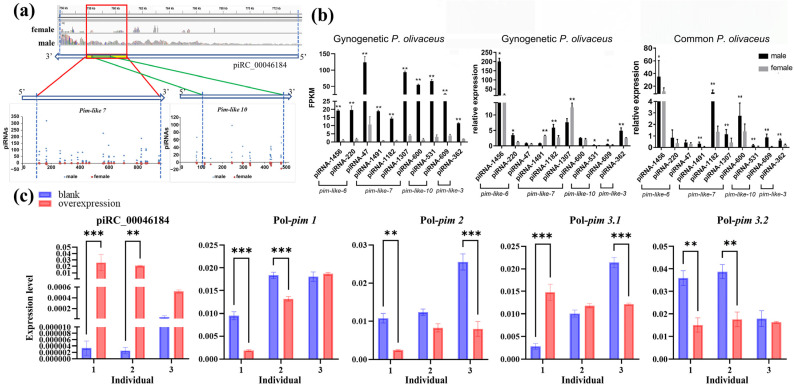
Regulation of piRNAs on the *pim* genes. (**a**) The mapped piRNAs from piRC_00046184 on the *pim-like 7* and *pim-like 10* genes. The red dots are piRNAs from ovaries and the blue dots are piRNAs from testes; (**b**) expression of piRNAs in *P. olivaceus* gonads as transcriptome data of gynogenetic *P. olivaceus*, qRT-PCR of common and gynogenetic *P. olivaceus* gonads; (**c**) qRT-PCR of *pim* genes after overexpression of piRC_00046184 in *P. olilvaceus* testes. Blank indicates untransfected testis tissues, * indicates *p* < 0.05, ** indicates *p* < 0.01, *** indicates *p* < 0.005.

To reveal the regulatory function of piRNA clusters on *pim* genes, the regulatory pattern was annotated for the piRC_00046184 and *pim-like 7*/*like 10* genes (Figure 6c). The results showed that these two expanded *pim*s are located within piRC_00046184, in reverse-complement orientation, and mapped to multiple piRNAs (Figure 7a). Expression analysis revealed that most piRNAs mapping to *pim-like* genes displayed male-biased expression (Figure 7b). To assess their regulatory effect on conventional *pim* genes, the overexpression plasmid pEGFP-piRC184 was constructed and transfected into *P. olivaceus* testes in vitro. At 72 h post transfection, piRC184 expression was markedly up-regulated, confirming successful overexpression (Figure 7c). Meanwhile, the expression of conventional *pim1*, *pim2*, and *pim3.2* was significantly down-regulated, indicating that piRC184 overexpression can influence the expression of conventional *pim* members (Figure 7c). These results supported the notion that expanded *pim* genes in *P. olivaceus* may act as piRNA cluster templates, generating piRNAs that regulate the expression of conventional *pim* genes.

## 4. Discussion

### 4.1. Specific Characteristics of PIWI/piRNA Pathway in Common and Gynogenetic P. olivaceus

piRNAs associate with PIWI proteins to form piRNA complexes, which are primarily expressed and function within the germline of animals. PIWI/piRNA pathway genes are predominantly expressed in the gonads, with high expression of *piwi* genes reported in mouse testes, where they play critical roles in spermatogenesis [49]. In *P. olivaceus*, PIWI/piRNA pathway genes exhibit a similar expression pattern, with most showing strong testis-biased expression in common *P. olivaceus*, consistent with observations in other species [50]. During development, these genes are mainly expressed in early embryogenesis, with expression levels decreasing after hatching, showing a similar trend to that observed in *Takifugu fasciatus* [51]. In *P. olivaceus*, the testes and early embryos exhibited piRNA enrichment organized into clusters. In oysters, large numbers of piRNAs were identified in the gonads, showing strong ping-pong amplification signatures, with piRNA clusters specifically expressed during the first 20 days post fertilization [34]. In the yellow fever mosquito (*Aedes aegypti*), piRNAs were also abundantly expressed during embryogenesis [52]. In the tongue sole (*Cynoglossus semilaevis*), comparative analyses revealed abundant piRNA expression in both males and pseudo-males, particularly in testes [53]. These findings collectively suggest that the PIWI/piRNA pathway in common *P. olivaceus* is conserved across species both in the tissues and developmental stages.

Gynogenesis induction has been widely adopted in fish biology research and aquaculture, and in *P. olivaceus*, where females grow larger and faster, it offers a valuable strategy to enhance economic returns. However, gynogenetic *P. olivaceus* frequently exhibit low fertilization rates, impaired gonadal development, and reduced gamete quality [20], presenting major hurdles for its practical application. In this study, PIWI/piRNA pathway genes in gynogenetic *P. olivaceus* displayed testis-biased expression patterns similar to those in common *P. olivaceus*. Strikingly, the ovaries of gynogenetic *P. olivaceus* contained abundant piRNAs organized into clusters comparable to those in the testes. Analogous observations of high piRNA levels in *Drosophila* ovaries and the functional impacts of piRNAs on mouse ovarian development indicated that piRNAs also act in female germ cells [17,18], potentially contributing to the gonadal and gametogenic defects seen in gynogenetic *P. olivaceus*. Moreover, ovarian piRNA clusters in gynogenetic *P. olivaceus* targeted the *LINE/L2*, *LINE/Rex-Babar*, and *LTR/Gypsy* transposon families, mirroring the transposon targets of testicular piRNA clusters in common *P. olivaceus*, and echoing findings in oysters, where piRNA clusters likewise target *LINE/L2* and *LTR/Gypsy* elements [34]. Early functional studies demonstrated that piRNAs preferentially recognize and silence retrotransposons (e.g., *LINE1*) to safeguard genome integrity [54]. Our results further corroborate a conserved role for the PIWI/piRNA pathway in targeting retrotransposons in *P. olivaceus*.

### 4.2. piRNA Targeting TEs in P. olivaceus

piRNAs recognize and silence transposon sequences to preserve genome integrity and stability [13]. *P. olivaceus* piRNA clusters target numerous transposon families, including *LINE/L2*, *LINE/Rex-Babar*, and *LTR/Gypsy*, suggesting a regulatory role for piRNAs in transposon repression. In mice, piRNAs have been shown to silence numerous retrotransposons, such as *LINE1* [55]. In the sea urchin, they targeted *Harbinger* and *SINE2-1* elements [56]. In medaka and swordtail testes, piRNA clusters map to *LINEs*, *SINEs*, and various DNA transposons [57]. These targeting profiles closely mirror those observations in *P. olivaceus*. The *Tc1/Mariner* superfamily of DNA transposons is broadly distributed across bacteria, invertebrates, and vertebrates [58] and is particularly prevalent in teleosts [59]. To maintain genomic stability, many transposons have accumulated structural defects during evolution, rendering them inactive. Indeed, in mammals, most *Tc1/Mariner* family members are deletion derivatives lacking transposition activity [60]. In *P. olivaceus*, we identified nine *Tc1/Mariner* family elements that phylogenetically cluster into five subfamilies, the majority of which are structurally incomplete, only two elements encode intact transposases. qRT-PCR demonstrated significant male-biased expression of these *Tc1* members in both common and gynogenetic *P. olivaceus*. Moreover, *Tc1* elements are the transposon family most heavily targeted by piRNAs in gynogenetic *P. olivaceus*, suggesting a potential regulatory interplay and indicating that piRNA-mediated control in teleosts may extend beyond retrotransposons.

### 4.3. The Expanded Pim Family and piRNAs in P. olivaceus

*Pim* is an oncogene of the serine/threonine protein kinase family, known to regulate the cell cycle and apoptosis via phosphorylation of target proteins [61,62] and to promote cancer cell growth [63]. While few studies have linked *pim* to piRNA-mediated regulation, our results in *P. olivaceus* showed that many piRNAs target *pim* family members, consistent with observations in tilapia [64]. This suggested a potential piRNA-based regulatory mechanism for *pim*s in teleosts. In *P. olivaceus*, 14 members of the *pim* genes were identified, whereas mammals possess only three conventional *pim* genes. Beyond *P. olivaceus*, many other teleosts also exhibit expansion of the *pim* family, with clear divergence observed in phylogeny and sequence similarity. Considering the number of expanded genes, this expansion is unlikely to result solely from genome duplication in teleost [65], but may instead reflect functional diversification. In terms of expression, most *pim* genes in *P. olivaceus* and their targeted piRNAs exhibit pronounced male-biased expression, suggesting that these expanded *pim* genes may be functionally linked to the PIWI/piRNA pathway. Studies have shown that piRNAs can be transcribed using pseudogenes as templates to regulate other members of the same gene family [66]. In *P. olivaceus*, the expanded *pim*-like 7 and *pim*-like 10 genes overlapped with a long non-coding RNA (piRC_00046184) in a reverse-complement orientation, suggesting that these expanded *pim* genes may act as pseudogenes, serving as templates for piRC184-mediated piRNA transcription. However, some studies argued that piRNA biogenesis is largely independent of pseudogenes, implying that these pseudogenes are merely byproducts of piRNA cluster formation [67]. Our results are more consistent with the former scenario. Upon overexpression of piRC184, the expression of conventional *pim* members was largely down-regulated, indicating that the expanded *pim-like 7* and *pim*-*like 10* may function as auxiliary pseudogenes, facilitating the transcription of piRNAs complementary to *pim* sequences, which, in association with PIWI proteins, regulate the expression of other members of the *pim* family in *P. olivaceus.*

## 5. Conclusions

The PIWI/piRNA pathway was characterized in common and gynogenetic *P. olivaceus*, and clustered piRNAs were identified in the testes and early embryos of common *P. olivaceus*, consistent with patterns reported in other organisms. By contrast, the ovaries of gynogenetic *P. olivaceus* harbored abundant testis-like piRNA clusters, representing a marked divergence from common *P. olivaceus* and suggesting that piRNA-mediated regulation may play a distinct role in the gynogenetic lineage. In gynogenetic *P. olivaceus*, *pim* genes were heavily targeted by piRNAs, exhibited testis-biased expression and conspicuous expansion across teleosts. Expanded *pim* genes were adjacent to or overlapping with piRNA clusters, which could significantly reduce the expression of conventional *pim* genes, supporting the model that expanded pseudogene-like *pim*s may act as piRNA sources to regulate conventional *pim* members. These findings provide a foundation for mechanistic dissection of the PIWI/piRNA pathway in gynogenetic *P. olivaceus* and have important theoretical and practical implications for gynogenetic breeding.

## Data Availability

The datasets generated for this study can be found in the NCBI Sequence Read Archive (SRA) BioProject PRJNA764760 for gynogenetic *P. olivaceus* gonads. The datasets for common diploid *P. olivaceus* tissues and developmental stages are not readily available because the data are part of an ongoing study, and requests to access these datasets could be directed to the corresponding author Jie Cheng.

## Supplementary Materials

The following supporting information can be downloaded at: https://www.mdpi.com/article/10.3390/biology14101464/s1, Table S1: Primers used in this study; Table S2: Summarized information for piRNA clusters; Table S3: Transposons identified in *P. olivacues* genome; Table S4: *Tc1/Mariner* members identified in *P. olivaceus*. Table S5: Gene-targeted piRNAs in gynogenetic *P. olivaceus*.
