# Supplementary material for: Dynamic Regulation of Gonadal Transposons and Pseudogenes via PIWI/piRNA Pathway in Gynogenetic Japanese Flounder (Paralichthys olivaceus)
Source: Biology (Basel). 2025 Oct 21;14(10):1464. doi: 10.3390/biology14101464 (PMC12562241; doi:10.3390/biology14101464)
Supplement: Supplementary file 1 [file biology-14-01464-s001.zip › Table S1.Primers used in this study.pdf]

Table S1. Primer used in this study

Genes/Small-RNAs	Primer sequence (5'-3')
Tc1_1_Minos_Pol-F	GGCTACAACTGTCGCATTCCT
Tc1_1_Minos_Pol-R	GCGTCTCCACTCTTCCTCCA
Tc1_5_Bari_Pol-F	CTTTCTCAACGGAGGGAATGA
Tc1_5_Bari_Pol-R	TGTCTGAACCACGCCCACT
Tc1_6_Frog Prince_Pol-F	ACAGCAGCAGAAGACCAAACC
Tc1_6_Frog Prince_Pol-R	ACACCAACAGAAGCCTGAACC
Tc1_7_Passport_Pol-F	CTGCTCATCACCTGCCCAATA
Tc1_7_Passport_Pol-R	CAACCAGTCGTCCTGTCCCT
Tc1_9_pogo_Pol-F	AACTACCGTCTCGCAGCAACT
Tc1_9_pogo_Pol-R	GCGCTCTGGCCGGATC
pim1-F	CACTGAAGGGCACACAGATTA
pim1-R	CAAACACACACACGCACATC
pim2-F	GCGGCGACAGAAACTAATA
pim2-R	GGGACCTCAGATGTTGATTT
pim3.1-F	GGGCTTTCGTCAACAACT
pim3.1-R	GGAGAGGAGGAGAGAAACAT
pim3.2-F	TCAAGCAGGTCTCCATACA
pim3.2-R	GACAGAGAGCGAGACATAGA
pim-like-3-F	GGCCACCAAGGGAAAGG
pim-like-3-R	GACCGATGAGGAAAGAGAATCTG
pim-like-6-F	AACTGAACTTGTGCTCGAC
pim-like-6-R	CCTCCCATCTCACAATATGAC
pim-like-7-F	TGGTCCAACCACCGTAT
pim-like-7-R	GCGTCTGGTCTAACTTCAAA
piRNA-1182-F	TGAAATATTTGTCTCTTCTCCAGCC
piRNA-1307-F	TGAGAAAACTTCCAGTAGTGAAAACC
piRNA-1456-F	TGCGCTTTAGAAATTTCCTGAGGGT
piRNA-1491-F	TGGACTCAATGGTGGGAGGAGATTTT
piRNA-220-F	TAATGTTTGTCTTCCAAATAGCAGC
piRNA-362-F	TAGACTGATCCAAAGCCTCCTTCAC
piRNA-47-F	TAAAGATTGGCTCGCCCTCTGGGATC
piRNA-531-F	TATTTATCCAGGAACTTAGCTCTTGCT
piRNA-600-F	TCAACCATGGGTGATGCTGGAGCTCT
piRNA-609-F	TCAACTCGTTGCTCAGGTCATACCAGTC
pEGFP-piRC184-F	GGACTCAGATCTCGACATTCGTCTTCCTGATGAGCC
pEGFP-piRC184-R	CCGCGCTACCGTCGAATTGCCCTGTCACAGTTTTGCC
miR-22-3p	AAGCTCAGCTGAGAACTGT
miR-23a-3p	ATCACATTGCCAGGGATTTCCA
ACTB-F	CCACCGCAAATGCTTCTA
ACTB-R	ACTGTCTCCATCGTTCCA
UBCE-F	TTACTGTCCATTTCCCCACTGAC
UBCE-R	GACCACTGCGACCTCAAGATG

Notes: F for forward primer, R for reverse primer.
