# Supplementary material for: Dynamic Regulation of Gonadal Transposons and Pseudogenes via PIWI/piRNA Pathway in Gynogenetic Japanese Flounder (Paralichthys olivaceus)
Source: Biology (Basel). 2025 Oct 21;14(10):1464. doi: 10.3390/biology14101464 (PMC12562241; doi:10.3390/biology14101464)
Supplement: Supplementary file 1 [file biology-14-01464-s001.zip › Table S3.Transposons identified in Pol genome.pdf]

Table S3. Transposons identified in *P. olivaceus* genome

Class	Family	Sequence	Copy	Total
LINE	L2	83	29697	44858
	Rex-Babar	37	9247	
	R2-Hero	8	2276	
	Penelope	6	1808	
	I	3	1416	
	Dong-R4	1	226	
	Proto2	1	188	
SINE	SINE	7	4666	17428
	ID	1	57	
	MIR	5	2963	
	tRNA-Core	3	4759	
	tRNA-Core-L2	1	956	
	tRNA-Core-RT	1	344	
	tRNA-L1	2	868	
	tRNA-V	4	2497	
LTR	Gypsy	20	20943	26075
	Ngaro	4	4853	
	LTR	1	189	
	ERV1	1	90	
DNA	hAT-Ac	45	27579	626541
	DNA	34	27305	
	P	22	26425	
	PIF-Harbinger	43	20466	
	Zisupton	3	13686	
	CMC-EnSpm	13	12088	
	TcMar-Tc1	34	11104	
	hAT-Charlie	23	7779	
	hAT-Tip100	9	7280	
	Kolobok-T2	4	6812	
	Crypton-V	10	6603	
	Kolobok	3	6030	

	hAT-hAT5	4	4263	
	hAT	4	2157	
	hAT-Blackjack	2	620	
	TcMar-Fot1	2	516	
	Sola-2	1	422	
	PiggyBac	1	408	
	TcMar-Tc2	1	192	
	TcMar-Pogo	1	168	
	hAT-hobo	1	164	
	RC/Helitron	29	444474	
Simple repeat	Simple_repeat	2	662	2301
	Satellite	4	1639	
multicopygene	rRNA	1	286	286
