# Supplementary material for: Dynamic Regulation of Gonadal Transposons and Pseudogenes via PIWI/piRNA Pathway in Gynogenetic Japanese Flounder (Paralichthys olivaceus)
Source: Biology (Basel). 2025 Oct 21;14(10):1464. doi: 10.3390/biology14101464 (PMC12562241; doi:10.3390/biology14101464)
Supplement: Supplementary file 1 [file biology-14-01464-s001.zip › Table S4.Tc1-Mariner members identified in Pol.pdf]

Table S4. *Tc1/mariner* members identified in *P. olivaceus* genome

ID	Name	Family/subfamily	Length (nt/aa)	ITR end	Catalytic motif
NW_017859675.1:749673-752257	Tc1_1_Minos_Pol	Minos-like	744 / 247	not found	DD34K
CM007771.1:12993655-12996266	Tc1_2_Minos_Pol	Minos-like	612 / 203	not found	DD34E
NW_017860625.1:12730-15467	Tc1_3_Minos_Pol	Minos-like	738 / 245	not found	DD34E
NW_017859707.1:591892-593733	Tc1_4_Bari_Pol	Bari-like	669 / 222	not found	DD34K
NW_017859657.1:9251618-9254475	Tc1_5_Bari_Pol	Bari-like	858 / 285	not found	DD34D
NW_017859792.1:293372-296277	Tc1_6_Frog Prince_Pol	Frog Prince-like	1023 / 340	CAGTG	DD34E
NW_017859718.1:597974-600657	Tc1_7_Passport_Pol	Passport-like	981 / 326	CAGTG	DD34E
NW_017859649.1:3197623-3200366	Tc1_8_Passport_Pol	Passport-like	744 / 247	CAGTG	DD34E
NW_017859675.1:20035-22880	Tc1_9_pogo_Pol	pogo-like	846 / 281	not found	DD35D
